# Structural Violence and Health-Related Outcomes in Europe: A Descriptive Systematic Review

**DOI:** 10.3390/ijerph18136998

**Published:** 2021-06-30

**Authors:** Gloria Macassa, Cormac McGrath, Mamunur Rashid, Joaquim Soares

**Affiliations:** 1 Department of Public Health and Sports Science, Faculty of Occupational and Health Sciences, University of Gävle, Kungsbacksvägen 47, 80176 Gävle, Sweden; Mamunur.Rashid@hig.se; 2 EPIUnit–Instituto de Saude Publica, Universidade do Porto, Rua das Taipas 135, 4050-600 Porto, Portugal; 3 Department of Education, Stockholm University, Frescativägen 54, 10691 Stockholm, Sweden; cormac.mcgrath@edu.su.se; 4 Department of LIME, Karolinska Institutet, Solnavägen 1, 17177 Solna, Sweden; 5 Department of Health Sciences, Mid-Sweden University, Holmgatan 10, SE-85170 Sundsvall, Sweden; joaquim.soares@miun.se

**Keywords:** structural violence, health outcomes, social determinants of health, Europe

## Abstract

In recent years, there has been a revival of the term “structural violence (SV)” which was coined by Johan Galtung in the 1960s in the context of Peace Studies. “Structural violence” refers to social structures—economic, legal, political, religious, and cultural—that prevent individuals, groups and societies from reaching their full potential. In the European context, very few studies have investigated health and well-being using an SV perspective. Therefore, this paper sought to systematically and descriptively review studies that used an SV framework to examine health-related outcomes across European countries. The review included two studies each from Spain and France, one each from the UK, Ukraine and Russia, and another study including the three countries Sweden, Portugal and Germany. With the exception of one mixed-method study, the studies used a qualitative design. Furthermore, the eight studies in the review used different conceptualizations of SV, which indicates the complexity of using SV as a concept in public health in the European context. Future research that attempts to identify and standardize measures of SV is needed; the knowledge gained is hoped to inform appropriate interventions aiming to reduce the effects of SV on population health.

## 1. Introduction

In recent years, there has been a revival of the term “structural violence (SV)”, which was first coined by Johan Galtung in the 1960s in the context of Peace Studies [1]. The term “structural violence” refers to the social structures—economic, legal, political, religious and cultural—that prevent individuals, groups and societies from reaching their full potential [1]. Galtung argued that these arrangements are “structural” because they are embedded in the political and economic organization of our social world; and “violent” because they are likely to cause injury to people—typically not those responsible for perpetuating such inequalities [1]. Others argue that the terminology attempts to give weight to how the effects of SV are to some extent an “impairment to human life needs” [2], which would prevent someone from meeting their needs. In other words, SV lowers the degree to which they are able to meet their needs “below what would otherwise be possible” [2]. According to Gilligan, SV is mostly invisible and embedded in longstanding “ubiquitous social structures, normalized by stable institutions and regular experience” (e.g., resulting in differential access to resources, political power, education, and health care) [2]. In addition, Farmer argued that SV is closely linked to social injustice as well as to the social machinery of oppression [3].

Although SV has drawn research attention in the fields of sociology and anthropology, it has only in recent years been brought into the research discourse of the health sciences and, specifically, the public health sciences. According to De Maio and Ansell [4], the potential of SV theory in studying health outcomes lies in its focus on deeper structural roots of health inequalities rather than in it being a passive approach centered on the social determinants of health (SDHs), i.e., a social epidemiological approach [4]. Traditionally, the social epidemiological approach identifies social characteristics that affect the pattern of disease and health distribution in a society in order to understand its mechanisms [5,6,7].

The concept of SV has similarities with the concept of “structural determinants of health” (a term coined by the World Health Organization (WHO) Commission on the Social Determinants of Health (CSDH)) [8]. The term “structural determinants of health” refers to mechanisms that generate stratification and social class divisions in society and that simultaneously define an individual’s socioeconomic position in terms of “hierarchies of power, prestige and access to resources” [8]. De Maio and Ansell [4] argue that the terms “SV” and “structural determinants of health” call attention to the societal arrangements that exist upstream from the “behaviour and biology of individuals; they both extend the traditional social determinants of health model by prioritizing the causal force of structural forces” [4]. However, SV has a distinct etiology, as it describes health inequalities as an act of violence, arguably adding something that the “structural determinants of health” term lacks. For instance, it is suggested that the SV approach is capable of revealing dynamics of social practices that operate across multiple dimensions of people’s lives in ways that may not immediately appear to be related to health [3,9]. Furthermore, SV takes into consideration the extent to which people’s lives are affected by institutionalized inequality, influencing and often governing individual experience [3,9]. De Maio and Ansell went further to suggest that SV identifies in an explicit way the social, economic and political system as the “causes” of poor health.

There still is an ongoing debate on what precise aspects should be measured to enable the use of SV theory in social epidemiology [4]. Research using the SV approach has increased over the past 30 years, especially in the Global South (low and middle-income countries) as compared with the Global North (mostly the US and Canada). It is argued that the extensive use of the SV lens in the Global South has been fueled by the need to better understand historical and political trauma, gender inequality, and poverty [10,11,12,13,14,15].

Combining different strategies (fieldwork, analyses of public policy documents, observation, and interviews with indigenous peoples and managers), Teixeira and Da Silva attempted to establish correlations between interpersonal violence and SV along democratic processes of public policies building in Indigenous health care [14]. In their study, they proposed that SV in health needed to be interpreted against the backdrop of a broader discussion on the construction of Indigenous citizenship that included tutelage and political participation in the politics of health practices in Brazil [14]. In a study carried out in Sub-Saharan Africa, Joseph used community and country-level inequalities in gender relations, human rights violations, and globalization as markers of SV, and related them to maternal health care [15]. The results of the study indicate that inequalities in gender relations and disrespect for human rights were negatively associated with adequate use of maternal health care in Sub-Saharan Africa. In addition, social globalization was the most significant predictor of adequate maternal health care [15]. In Zimbabwe, using qualitative methods, Muderedzi and colleagues investigated how SV affected families of children with cerebral palsy among the Tonga ethnic group living in poor rural communities of Binga [13]. They identified SV through four themes: internal displacement and development; food and politics; water and sanitation; and social services. Structural violence was perceived to inflict social suffering on the study participants [13] and politics was reported to play a major role in activities such as food withdrawal, lack of water, development, and allocation of local resources to city residents, leaving the rural participants struggling for care [13].

In the Global North (i.e., high-income countries), research applying the SV lens to study health outcomes has addressed the situation of immigrants and other disadvantaged societal groups [16,17,18,19,20,21,22,23]. In the US, in a study investigating how fear among Hispanic migrants undermined the risk for diabetes, Page-Reeves et al. demonstrated that structural forces directly inhibited access to appropriate health care services and created fear among immigrants, further undermining health and nurturing disparity [18]. The authors observed that although fear was not directly associated with diabetes, participants nevertheless felt that there was a connection to their health outcomes [18]. Additionally, in the US, Saleem and co-authors used the SV framework to assess health and well-being outcomes among immigrants as well as LGBTQ+ persons [21]. Furthermore, in a study that explored the impact of access to health care on the lives of at-risk populations in Florida, Mead found that factors such as finances, mental health needs, personal issues, and lack of childcare prevented patients from accessing health care. This was despite the safety net programs that were in place to serve at-risk populations (e.g., those with a low income, as well as rural and minority populations) [22].

Using the SV framework, Banerjee et al. [23] found that, compared with their Scandinavian counterparts (in Denmark, Norway and Sweden), Canadian frontline care workers reported higher rates of violence [23]. The participants in their study reported structural factors such as insufficient staff, heavy workload, lack of decision-making autonomy, inadequate relational care and rigid work routines [23].

In the European context, however, very few studies have investigated health and well-being using an SV perspective. This lack of research is set against a backdrop of increasing reports of structural violence experienced by certain groups throughout the continent (e.g., sexual workers, ethnic minorities, etc.) [24,25,26,27]. Therefore, we sought to review studies that used an SV framework to examine health-related outcomes across European countries. The specific objectives were to examine: (a) what types of studies (in terms of design) were conducted and in which countries they were carried out; and (b) what dimensions were used to conceptualize SV across the identified studies.

## 2. Materials and Methods

### 2.1. Search Strategy

A systematic literature search was carried out using Medline and Web of Science. Two trained librarians working at Karolinska Institutet, Stockholm, with experience in systematic reviews carried out the search according to the Preferred Reporting Items for Systematic Reviews and Meta-Analyses (PRISMA) guidelines [28]. The searches had no time limit. Peer-reviewed, English-language empirical research articles were included if the research had been conducted in Europe and if they addressed the relationship between SV and “any type of health outcomes”. The words “structural violence” needed to appear in the publication title or abstract or key words, and the research needed to have been framed through an “SV theory lens”. Publications were excluded if they were editorials, preprints, letters, reviews, and theory-building research without empirical data.

The following key search terms were used: “structural violence and health in Europe”, “structural violence and any disease in Europe”, “structural violence and well-being in Europe”, “structural violence and health care in Europe”, “structural violence and physical health in Europe”, “structural violence and psychological health in Europe”, and “structural violence, intimate partner, and physical and psychological health in Europe”.

### 2.2. Article Selection and Assessment

**Figure 1:**
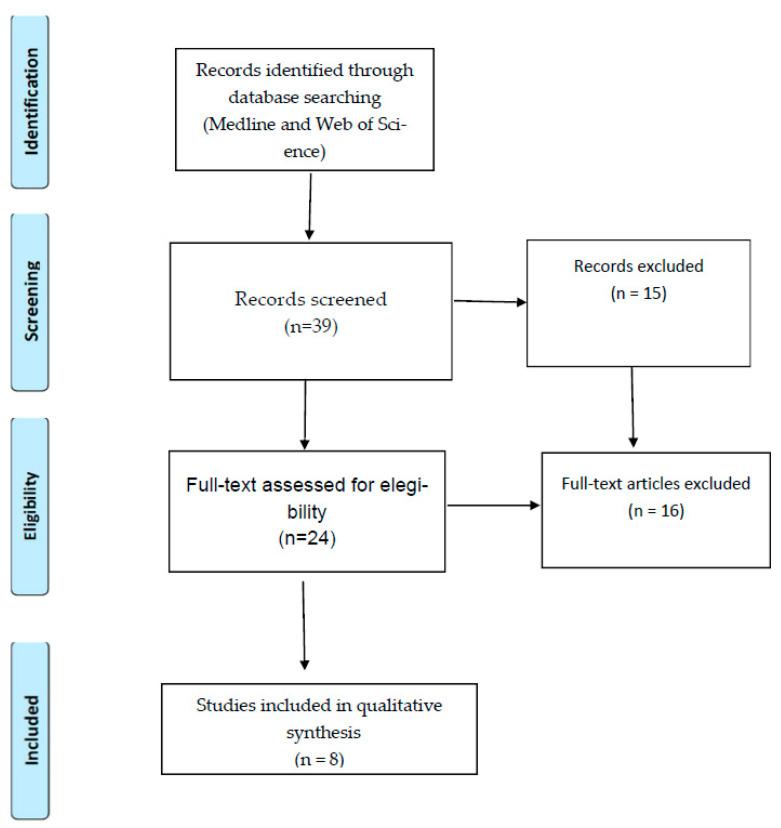
Identification, screening, and inclusion of studies for the review according to PRISMA.

The search carried out in Medline and Web of Science identified 39 articles after removing duplicates. The articles were exported to Mendeley Reference Management Software [29] for a process of manual screening that was undertaken in two steps. In the first step, the authors G.M., C.M. and M.R. removed 15 articles (e.g., conceptual articles and reviews). In the second step, the full text of the remaining 24 articles was assessed for eligibility. Of these articles, 16 were further excluded because they did not include SV conceptualization or had unclear methodology. Potential disagreements regarding inclusion and exclusion criteria were resolved between the four co-authors (G.M., C.M., M.R., and J.S.), taking into account the relevance of the publications and the research question of this review. Eight articles met the inclusion criteria (see Figure 1).

## 3. Results

This section presents the characteristics (in terms of country of origin, and design) of the studies included in the review as well as descriptions of the conceptualization of SV, and key findings for each of the studies.

### 3.1. Characteristics of the Included Studies

The eight studies that met the inclusion criteria were published between 2010 and 2021. Two were from Spain [30,31] and two from France [32,33], and one each was from Ukraine [34], Russia [35], the three countries Sweden, Portugal, and Germany [36], and the UK [37]. Seven studies used a qualitative method design; one study used a mixed (qualitative and quantitative) design. Sample size varied from 5 [37] to 209 interviewees [35] (see Table 1).

### 3.2. Conceptualization of Structural Violence and Key Findings across the Reviewed Studies

The Sánchez-Sauco et al. study (*n* = 10) used a socio-cultural approach (including socio-economic status) to examine socio-cultural influences on perinatal drug dependency among pregnant women. The results indicate that there was criminalization and stigmatization associated with addiction, and that the women experienced multi-layered social barriers when seeking rehabilitation services [31]. Similarly in Spain, the study by Rodríguez-Martínez and Cuenca-Piqueras (*n* = 32) investigated how sexual harassment in the workplace intersected with other forms of direct and indirect violence among undocumented migrant women who were domestic and sex workers. The results indicate that the interviewed women did not consider verbal abuse as sexual harassment and attributed the abuse to their work. Furthermore, interviewees perceived sexual harassment to be due to of lack respect. The study also indicated that sexual harassment had fewer negative consequences for women compared with intimate partner violence [30].

In a small study (*n* = 5) in France, Larchanché investigated the obstacles experienced by undocumented migrants in realizing their health care rights. The findings pointed out that while, legally, undocumented immigrants are entitled to health care in France, the consequences of their social stigmatization and of their precarious living conditions, and the climate of fear and suspicion generated by increasingly restrictive immigration policies in practice prevent many from feeling entitled to that right [32]. In another French study, Pursch and colleagues studied how non-state providers’ policies affected health service provision to migrants (*n* = 20) in Calais and La Linière in northern France [33]. The study found that the role of non-governmental organizations (NGOs) in providing migrant health services in northern France was complex and contested. There were indications that SV negatively affected migrant’s well-being through restricted services, intentional chaos, and related disempowerment. The SV exerted on migrants appeared to diminish their life chances. In addition, NGOs were required to adapt service delivery to fit within the boundaries set by the government, such as limiting distribution points to one hour and constantly changing their location to ensure that individuals living on the streets were less able to access services [33].

In Ukraine, Owczarzak et al. investigated how red-tape bureaucracy and paperwork were a form of SV in the provision of health services for female drug abusers (*n* = 37) [34]. The study indicated that documentation requirements were enacted as a form of SV towards already marginalized women (through use of coding for marginalized, stigmatized, ill and/or disabled identities) and prevented them from accessing the services and resources they needed. In addition, despite the benefits that official status could confer, both clients and providers criticized the system because it often excluded the very women who needed help the most [34].

The study by Hamed et al. (*n* = 11) was carried out in Germany, Portugal and Sweden and used racial discrimination as an analytical lens. It investigated accessibility to health care among users in the three countries. The included health care users felt that medical staff regarded their narratives as illegitimate and viewed them as unworthy of treatment; the study concluded that this was a form of SV [36].

In the UK, the Lewis and Russel study investigated the experiences of young smokers (*n* = 5)—both active smokers and those who were trying to quit smoking—at a youth club in alow-income neighbourhood [37]. The findings indicate that young people were somewhat caught between three competing domains: economic and political structures, media structures, and organized crime. These three domains together conspired to provide young people with means of consumption from which they were excluded through legitimate structures. The authors pointed out that, rather than expecting young people to act in accordance with the health risk advice, interventions were needed to bridge issues of agency and critical consciousness that could otherwise be eroded by SV [37].

The study by Sarang and colleagues investigated accounts of HIV risk and other health risks among drug users in Russia (*n* = 209) taking into account policing practices [35]. The study found that policing practices violated rights of drug users directly, but also indirectly, through inflicting social suffering. In addition, the study indicated that extrajudicial policing practices introduced fear and terror into the day-to-day lives of drug injectors. The fear and terror experiences ranged from the mundane (arrest without legal justification or evidence, in order to expedite arrest or detainment; extortion of money or drugs for police gain) to the extreme (physical violence as a means of facilitating confession, or as an act of “moral punishment” without legal cause or rationale; as well as torture and rape) [35].

## 4. Discussion

The majority of the studies included in this review used a qualitative design, which precludes exploratory analysis of these studies. Even in the study using mixed methodology, the main findings pertained to qualitative data. The same pattern was found among the empirical studies carried out outside the EU (two hundred and thirty-eight studies found in an extra search of studies published outside Europe), the majority of which used qualitative methodology (e.g., ethnographic and anthropologic designs), although there were some quantitative and mixed-method studies (e.g., reviews, case studies and policy analysis studies).

According to De Maio and Ansell, there is a challenge in conceptualizing SV, and specifically in spelling out the precise aspects of SV to be researched, which therefore poses difficulties for those interested in carrying out quantitative studies [4].

The review findings reported here also indicate that there was no uniformity in the theorization of SV, which in itself is why the studies in the review mostly used a qualitative design. The majority of studies conceptualized SV in terms of discrimination and racism [32,33,34,36] but socio-cultural status and community deprivation [31,37], power relations in violent victimization [30] and policing strategies [35] were also part of the conceptualization.

Discrimination and racism are often associated with health care accessibility [38,39,40]. It has been argued that there is a systemic segregation of and discrimination towards individuals seeking care in relation to their ethnicity, race, migration and status. Segregation and discrimination are based on income (in countries where health care needs to be paid for) and are often due to the failure to deliver appropriate care to minority groups in comparison with host populations [40].

For instance, Hamed et al. [36] framed racism as a form of SV; in their study, unequal access to resources led to silencing of suffering, and inequalities in power led to the erosion of dignity among their interviewees. These results are in line with those reported by Page-Reeves and colleagues where structural forces directly inhibited access to adequate health care services and created fear among Hispanic immigrants at risk of diabetes in the US [18]. It is assumed that individual behaviour somewhat affected by structured and institutionalized inequality is likely to limit the ability of respondents to get appropriate care. For instance, Larchanché reported that institutional discrimination and racism dynamics made migrants feel intimidated and fearful of institutional health contexts and led them to downplay their real needs and concerns in an attempt to make themselves more deserving of assistance [32]. Belliveau [41] termed these types of behaviours “strategies of acquiescence” and argued that such behaviours allow immigrants to balance their needs with the realities of discriminative environments and exclusionary policies [41]. In the Ukraine, Owczarzak and colleagues saw documentation requirements (including the requirement to carry a passport or resident permit) as a form of SV because they constituted a main barrier for female drug users to access the services they deserved [30]. In a recent review of the relationship between racism and health, Williams and colleagues provided an “overview of the evidence linking the primary domains of racism (structural racism, cultural racism and individual level discrimination) to physical and mental health outcomes”. They further argued that structural racism is the most important way through which racism affects health [40].

Sanchéz-Sauco et al. conceptualized SV in socio-cultural terms [31] and it was argued that, from a socio-cultural perspective, some individuals or groups are likely to face additional health risks on account of cultural values set by the dominant majority. In such contexts, there is a risk of potential perpetuation of stigmatization, marginalization, loss or devaluation of language and cultural practices, as well as less accessibility to culturally appropriate services including health care [42]. Sanchez-Sauco and colleagues found that criminalization and stigmatization of addiction were social barriers experienced by drug-dependent women when seeking rehabilitation services [31]. According to some, cultural inequality does not create economic inequality but rather, widens and legitimizes it [43,44]. In addition, cultural micro-aggressions can occur through everyday occurrences that send denigrating messages to certain individuals because of their social status in society (e.g., minority status, poverty, or disabled or any marginalized status) [43,44]. One study in the review used community deprivation and the community’s lack of material resources as a proxy for SV [37]. As already mentioned, SV manifests itself through separation of those who have power and material resources and those who are disadvantaged and powerless [3,9]. The difference in power was reflected in the distribution of resources determining whether individuals have shelter, or deprivation and unemployment; income; access to care, etc.

In their study, Lewis and Russel reported that community deprivation (an important determinant of health) had an impact on the interventions carried out to help youth improve their struggle to quit smoking [37].

Rodríguez-Martínez and Cuenca-Piqueras conceptualized SV in terms of gender imbalances in power, as well as discrimination, and identified an intersection between sexual harassment and SV [30]. The interviewed women in their study perceived that the perpetrators’ power over them increased due to their (the perpetrators’) perception that they (the women) were working in denigrating jobs. Furthermore, within the interviewed sample, immigrant women appeared to experience a compounded discrimination that combined sexual harassment with xenophobia and racist behaviour [30]. It has been reported that SV creates fertile conditions for interpersonal violence and emboldens engendered forms of violence towards women in vulnerable social positions [45]. Additionally, studies addressing violence against women have mainly focused on the proximate determinants associated with violent acts per se, ignoring the potential role played by structural systems that might facilitate the occurrence of interpersonal violence [45].

Sarang and colleagues also viewed policing strategies as SV. They argued that policing practices violate the health and rights of drug users directly, but also indirectly through inflicting social suffering [37]. According to Galtung, law enforcement can constitute SV when it is “indirectly built into repressive social orders creating differences between potential and actual human self-realization” [46]. This would mean an avoidable impediment to an individual in meeting their own needs “below what would otherwise be possible” [2], which in turn might constitute a “violation of an individual’s human rights” [47]. For instance, in a study that investigated cannabis crackdowns, SV was found to have had an impact on drug users’ well-being [48]. In the study, crackdowns were associated with police brutality, confiscation of funds, drugs and belongings, as well as stigma, discrimination, arrest and incarceration, and affected the respondents’ livelihoods, health and well-being [48].

Overall, although the studies in this review operationalized SV differently, one common picture emerged from the studied samples. The studies’ respondents and interviewees experienced some sort of suffering and harm inflicted by structural arrangements in the studied contexts, which had an impact on their health and well-being including their ability to access appropriate services (e.g., health care, and social and rehabilitation services). Moreover, the findings of these studies imply that there were structural inequities that were unjust and unfair and that were to some degree ingrained in the everyday social and policy structures. This is in line with the view that SV is often invisible and embedded in the structures, and normalized by stable institutions as well as regular experiences [49,50,51]. Furthermore, it has been pointed out that SV leads to suffering and death as often does direct violence; however, the damage produced by SV is slower, more subtle, more common, and more difficult to repair [4]. Opotow posits that SV is gradual, imperceptible and diffused in society as “the way things are done”, including whose voice is systematically heard, or ignored, and who gets particular resources and who gets to go without them [49]. In addition, Hernandez and Galleta argued that SV blurs agency and that no one person directly injures another; those harmed may themselves be seen as responsible for their own debilitation [52].

In an attempt to elucidate how SV is invasive and longstanding, a study carried out in New Zealand [53], which qualitatively analyzed midwives’ experiences of providing maternity care to socially disadvantaged women, found that there were three mechanisms through which these women were exposed to SV. Firstly, the disadvantaged women were structurally disempowered through reduced access to agency, lack of opportunities, and inadequate provision of basic human needs. Secondly, social disadvantage exacerbated risks inequitably by increasing barriers to care, aggravating the impact of adverse life circumstances and causing chronic stress. Thirdly, the neoliberal system in place emphasized individual responsibility, which perpetuated these inequities [53]. Neoliberalism as a form of SV has been investigated in the Global South as part of a legacy of structural adjustment programs (including a strong market-based approach that emphasized deregulation, minimization of the State, privatization, and the emergence of individual responsibility) that were conducted in the 1980s and 1990s across Latin America and Africa [54]. These structural adjustment policies aimed at achieving macro-economic stabilization, reducing governments’ role in the economy, privatizing public assets, and reducing public expenditure [54]. It is argued that neoliberal policies in the context of structural adjustment programs caused retrenchment of the welfare dimension of the state, as this dimension was seen as an impediment to the optimal functioning of the markets [55]. In terms of health, retrenchment of welfare meant fewer, more expensive, less controlled, and lower-quality health care services [56]. Furthermore, in many instances, the process of the reduction in the welfare state moved responsibility for taking care of people to the free market, which resulted in differences in the level and quality of care individuals received [57].

Furthermore, in the Global South, colonialism (and neo-patrimonialism) is seen as a form of SV [58]. The view here is that “subaltern societies” throughout the world to some degree still bear the imprint of their colonial legacy and also still suffer from the consequences of processes that were set in motion by colonization. According to Vaidya, post-colonial states largely inherited bureaucratic, political and legal institutions from their colonial predecessors which have been carried into the post-colonial era. In such countries, the colonial formal, rational–legal, political and bureaucratic structures coexist with societal norms and practices that contradict and subvert these structures [58]. Neo-patrimonialism is manifested through personalized control of state institutions, hiring practices based on tribal or ethnic loyalties, rampant use of public resources for the personal ends of public office holders, and a general disregard for the principle of rational–legal authority [58].

In the Global North, neoliberal policies related to austerity programs have also been found in some European countries as a response to the most recent economic recession through a decrease in budget expenditure [59]. For instance, Karamessini reported that in Europe, the neoliberal offensive had a disruptive effect on social cohesion, as well as on people’s lives and morale. This especially affected the most vulnerable [60]. In a review of evidence on the impact of austerity policies on health in the UK and Europe, Stuckler and colleagues found evidence that these measures had an impact on the poor, as well as widening the inequalities in health [61]. Furthermore, other studies found that austerity measures had an impact on the most disadvantaged (e.g., those in precarious employment and housing and those with pre-existing health problems); they also impacted mental health (e.g., rising suicide rates) and health services (e.g., accessibility) [61,62,63,64].

### 4.1. Areas for Further Research

This review has identified some areas where further research is needed. To start with, we found very few peer-reviewed studies using the concept of SV to investigate health-related outcomes in the European context. In addition, the majority of studies seemed to come from the medical anthropology discipline, often exploring the effects of oppressive conditions on health inequalities. Currently in Europe, the majority of studies addressing inequalities in health outcomes use SDH frameworks, SDHs being a core concept in social epidemiology. Herrick and Bell argue that the SV and SDH concepts have similarities inasmuch as both point to the unequal distribution of power, to social injustice and suffering, and to the effects of these factors on people’s capacity to live healthy lives [65].

We argue that European public health researchers, specifically those in the area of social epidemiology, need to join the debate on how SV might influence health-related outcomes, including health care. However, we acknowledge that there is complexity in how SV should be conceptualized (and measured) in health research. This concern has been addressed by various scholars [4,65,66,67]. For instance, De Maio and Ansell suggest that SV needs to be seen as a complex concept that has a rich explanatory potential but at the same time is vague in its operationalization and lack of theoretical precision [4]. They further posit that, contrary to the notion of social determinants of health which is a central pillar of social epidemiology, SV focuses on roots of health inequalities that go much deeper (as SV attempts to identify social, economic and political systems as causes of poor health outcomes) [4]. This complexity is demonstrated by the variety of proxies that were used in each study included in this review. We agree with those who suggest that one way to improve SV measurement is to develop frameworks aimed at identifying the “structural determinants of health” as a way to collate the concepts of social determinants of health and SV [4]. This move would allow the integration of the two concepts and facilitate a dialogue between medical anthropologists and social epidemiologists [4]. Above all, because SV and SDHs place individuals and communities at different levels of suffering and health and well-being, they provide a greater opportunity for meaningful prevention at the systemic level [4,63].

### 4.2. Strengths and Limitations

This review provides one of the first assessments of empirical studies that used the SV lens to investigate health outcomes in the European context. In addition, broad search terms were used in the literature searches which identified articles that met the inclusion criteria. Moreover, the searches were conducted by professional librarians with vast expertise in the systematic review process. Nevertheless, the review has some limitations as it relied solely on peer-reviewed studies that were published in the English language in indexed databases. Therefore, it is possible that publications addressing SV and health outcomes written in other languages do exist, but these were not included.

## 5. Conclusions

This review sought to describe studies using an SV framework to investigate health-related outcomes in Europe in terms of: the country where they were carried out; the design; and how SV was operationalized. We found two studies each from Spain and France, one each from the UK, Ukraine, and Russia, and one final study performed in Sweden, Portugal and Germany. With the exception of one mixed-method study, the included studies used a qualitative design. Furthermore, the eight studies in the review used very different conceptualizations of SV, which indicates the complexity of using SV as a concept in public health in the European context. Future research is needed to identify and standardize measures of SV, which will be essential to inform appropriate interventions aiming to reduce the effects of SV on population health.

## Figures and Tables

**Table 1:** Studies included in the review (*n* = 8).

Author, Year/Country/Reference	Study Objective	Design, Sample, and Method of Analysis	Conceptualization of Structural Violence (SV)	Health-Related Outcome (s)	Findings
Sanchéz-Sauco, 2019/Spain/[31]	To contribute to closing the current gap in the literature that holistically examines socio-cultural influences on perinatal drug dependency.	Qualitative study (semi-structured interviews)/thematic analysis Perinatal substance use and/or drug dependency in 10 pregnant women.	Socio-cultural factors	Substance use/drug dependency	The criminalization and stigmatization of addiction, and the risk discourse elucidate the multi-layered social barriers that drug-dependent women experience when seeking rehabilitation services.
Rodríguez-Martínez and Cuenca-Piqueras, 2019/Spain/[30]	To investigate how sexual harassment in the workplace intersects with other forms of direct and indirect violence towards Spanish and unauthorized migrant women working in sex and domestic work who have suffered direct and indirect violence.	Qualitative study/multi-level intersectional analysis Interviews with 32 Spanish and unauthorized migrant women (Latin American, Eastern European, and African).	Power imbalance and discrimination (related to working as a sex worker and immigrant status)	Intimate partner violence/sexual harassment	Findings were that the interviewed women did not consider verbal abuse as sexual harassment and attributed the abuse to their work. In addition, they perceived sexual harassment to be linked to respect and not to love. The authors indicated that sexual harassment had less devastating consequences for women than did intimate partner violence.
Larchanché, 2012/France/[32]	To identify obstacles for undocumented immigrants to realize their health care rights.	Qualitative participant observation, critical review of legislative debates and reports related to health care of migrants (*n* = 5)/ethnographic analysis.	Social stigmatization, precarious living conditions, fear created by restrictive immigrant policies	Health care access	Findings showed that while, legally, undocumented immigrants were entitled to health care rights in France, the consequences of their social stigmatization and of their precarious living conditions, and the climate of fear and suspicion generated by increasingly restrictive immigration policies in practice hindered many from feeling entitled to those rights.
Pursch et al., 2020/France/[33]	To explore the provision of health services to migrants in Calais and La Linière in northern France; to contribute to the discourse on the effects of SV on non-governmental service providers and migrants in precarious conditions; and to inform service provision policies.	Qualitative (semi-structured interviews)/20 key interviewees—Non-governmental organization (NGO) representatives/thematic analysis.	Immigrant status	Health care access	Structural violence negatively affected migrant well-being through restricted services, intentional chaos, and related disempowerment. The NGOs were required to shift service delivery to adhere to boundaries set by the government, such as limiting distribution points and constantly changing distribution locations to ensure that individuals living on the streets had difficulties to access services.
Owczarzak et al., 2021/Ukraine/[34]	To explore paperwork as a form of SV through its production of “legitimate” citizens, often through reinforcement of gender stereotypes and moral narratives of deservingness. In addition, the study examined the relationship between the government and NGOs in the provision of services to women who used drugs.	Qualitative study including 78 participants (41 medical and social service providers and 37 women who used drugs)/grounded theory.	Paperwork bureaucracy	Health and social care provision	Documentation requirements were enacted as a form of SV towards already marginalized women through use of coding for marginalized, stigmatized, ill, or disabled identities, and prevented the women from accessing the services and resources they needed.
Hamed et al., 2020/Sweden, Germany and Portugal/[36]	To study access to health care in several neighbourhoods by interviewing local health care users.	Qualitative study (semi-structured interviews)/11 interviewees (health care users)/thematic analysis.	Discrimination (racism, racial inequalities)	Health care access	Findings were that users felt that medical staff viewed these patients’ narratives as illegitimate, and regarded the patients as unworthy of treatment, which often resulted in a delay in treatment.
Lewis and Russel, 2013/United Kingdom/[37]	To understand the issues faced by young smokers—and those trying to quit smoking—in a deprived community.	Qualitative study (ethnographic study with participant observation) including 5 members of a youth club located in a disadvantaged neighbourhood.	Neighbourhood deprivation	Smoking /quitting smoking	The study found that young people were somewhat caught between three competing domains (economic and political structures, media structures, and organized crime). These domains together conspired to provide young people with means of consumption from which they were excluded through legitimate structures.
Sarang et al., 2010/Russia/[35]	To explore accounts of HIV and health risks among injection drug users.	Mixed-method study including a qualitative (semi-structured interviews) and quantitative (descriptive) design and a sample of 209 injection drug users. Qualitative data analysed using thematic analysis.	Drug policing strategies	Drug use/risk of physical violence	The study found that policing practices violated the rights of drug users directly, but also indirectly, through inflicting social suffering. Extrajudicial policing practices introduced fear and terror into the day-to-day lives of drug injectors, and ranged from the mundane (arrest without legal justification or evidence in order to expedite arrest or detainment; and extortion of money or drugs for police gain) to the extreme (physical violence as a means of facilitating confession, and as an act of “moral punishment” without legal cause or rationale, as well as torture and rape).

Human immunodeficiency virus (HIV); Non-governmental organization (NGO); Structural violence (SV).

## Data Availability

Not applicable.
